# Sanitary Inspection

**Published:** 1885-04

**Authors:** 


SANITARY INSPECTION. "CLEANLINESS IS THE BEST PROTECTION AGAINST CHOLERA."

The system of house-to-house inspection inaugurated in this State by the State Board of Health, must result in good, even if but imperfectly carried out. It is very true that "knowledge of defects and evils is the first step toward their remedy," and the information gained by the laity by this inspection, in many parts of the State, will come as a revelation to a large number of people who have given little, if any, attention to the subject of sanitation. The efforts, so far, of sanitarians have been made chiefly where there are large aggregations of individuals. These numbers render the work the more important only because of the numbers. Each year efforts are being made to extend the field of this useful work more than in the past. It must result in an improved sanitary condition wherever such requirements as those of our Board of Health are enforced.

This improved condition will do much to reduce the amount of the ordinary preventable diseases, and the knowledge gained by this effort will be far-reaching in its effect by drawing attention to the importance of removing disease-breeding nuisances that have long been tolerated, in proximity to dwellings and work-shops, from ignorance of their deleterious effects, and also from the indifference which comes from being "seen too oft," and with the same evil results that is said to attend increasing familiarity with vice.

Irrespective of any outbreak of cholera or other epidemic disease, this inspection must prove a gain, but should cholera appear in this country during this year, which its past history renders probable, we shall have the responsibility resting upon ourselves if we fail to do our utmost to provide against it, after the warning we have had during the last year. In such an event, this house-to-house inspection, with its probable result of cleansing, will be of incalculable advantage in diminishing the evil results of such a visitation. Since the past history of cholera has shown that it is accustomed to follow the lines of human travel and traffic, thorough disinfection along the lines of travel, as far as this is practicable, has been urged. This is both wise and timely, and commends itself to the thoughtful and provident.

Our State Board of Health should receive the active support both of the medical profession and of the public in the laudable effort it is making to improve the sanitary condition of our people and of the State, and thus, by preventive measures, to prolong life and secure health, happiness and prosperity, if we should escape the threatened visitation, and, in case of its occurrence, to be as well prepared as possible to battle with it, and to reduce its evil results to the smallest practicable proportions.

